# Feasibility, design and conduct of a pragmatic randomized controlled trial to reduce overweight and obesity in children: The electronic games to aid motivation to exercise (eGAME) study

**DOI:** 10.1186/1471-2458-9-146

**Published:** 2009-05-19

**Authors:** Ralph Maddison, Louise Foley, Cliona Ni Mhurchu, Andrew Jull, Yannan Jiang, Harry Prapavessis, Anthony Rodgers, Stephen Vander Hoorn, Maea Hohepa, David Schaaf

**Affiliations:** 1 Clinical Trials Research Unit, University of Auckland, Private Bag 92019, Auckland 1142, New Zealand; 2 School of Kinesiology, University of Western Ontario, 1151 Richmond Street, London, Ontario, Canada, N6A 3K7; 3 The George Institute for International Health, PO Box M201, Missenden Rd, New South Wales 2050, Australia; 4 Sport and Recreation New Zealand, PO Box 2251, Wellington 6140, New Zealand; 5 Ministry of Pacific Island Affairs, PO Box 97005, South Auckland Mail Centre, Auckland 2240, New Zealand

## Abstract

**Background:**

Childhood obesity has reached epidemic proportions in developed countries. Sedentary screen-based activities such as video gaming are thought to displace active behaviors and are independently associated with obesity. Active video games, where players physically interact with images onscreen, may have utility as a novel intervention to increase physical activity and improve body composition in children. The aim of the Electronic Games to Aid Motivation to Exercise (eGAME) study is to determine the effects of an active video game intervention over 6 months on: body mass index (BMI), percent body fat, waist circumference, cardio-respiratory fitness, and physical activity levels in overweight children.

**Methods/Design:**

Three hundred and thirty participants aged 10–14 years will be randomized to receive either an active video game upgrade package or to a control group (no intervention).

**Discussion:**

An overview of the eGAME study is presented, providing an example of a large, pragmatic randomized controlled trial in a community setting. Reflection is offered on key issues encountered during the course of the study. In particular, investigation into the feasibility of the proposed intervention, as well as robust testing of proposed study procedures is a critical step prior to implementation of a large-scale trial.

**Trial registration:**

Australian New Zealand Clinical Trials Registry ACTRN12607000632493

## Background

Childhood obesity has reached epidemic proportions in developed countries [1]. In New Zealand (NZ) almost one third (31%) of children aged 5–14 years old are overweight or obese [2]. The causes of obesity are multi-factorial. Research evidence points toward an imbalance between energy intake (food consumed) and energy expenditure (physical activity) [3]. Current lifestyles and environments are thought to discourage regular physical activity and encourage sedentary behaviors in children [4,5]. In particular, sedentary screen-based behaviors (such as television watching, video game play, and computer use) are thought to displace physical activity and are independently associated with obesity [6] and other adverse health outcomes such as hypertension [7]. In NZ, television watching is the most popular leisure time activity [8]; data from multiple sources [2,9,10] show that NZ children watch more than two hours of television on average per day. Video game playing is also pervasive. A report from the United Kingdom found that 91% of children played three or more gaming formats (or platforms), with video console games the preferred choice. For 11–16 year old children, 74% played 3–7 times per week, with an average duration of 1.9 hours (per session). Similar results have been reported for 6–10 year olds [11]. This video game usage mirrors that seen in NZ and other countries [12,13]. A recent review [14] found a positive association between non-television screen viewing (e.g., video games, computer games) and obesity. Similarly, a positive association was reported between time spent playing screen games and obesity after controlling for television watching.

To date, interventions aimed at decreasing obesity in children have been largely unsuccessful. A recent Cochrane systematic review of trials to prevent obesity [15] found 22 studies; ten long-term (at least 12 months) and 12 short-term (12 weeks to 12 months) that investigated a variety of single or multifaceted interventions [16]. Of the long-term studies, six combined dietary and physical activity interventions [17-22]. Five of these studies resulted in no difference in overweight status between groups. Two long-term studies used physical activity as the sole intervention [23,24]. Of these, a multi-media approach appeared to be effective in preventing obesity. Four short-term studies used physical activity as the sole intervention [25-28], and two [25,28] resulted in minor reductions in overweight status in the intervention group. The other eight short-term studies combined advice on diet and physical activity, but none had a significant impact.

The authors of this review highlighted a number of significant design flaws which marred the majority of studies; 1) being underpowered and/or poorly designed given the complexity of the intervention and outcomes sought; 2) many were short term in duration; and 3) lack of environmental influence that would affect the sustainability of the intervention. Despite these limitations, the authors concluded that strategies to increase physical activity and reduce sedentary behavior may be fruitful in preventing overweight and obesity in schoolchildren and that further well-designed randomized controlled trials are required [16].

Turning off television has been advocated as a population health strategy [10,28]. However, television and computer games offer a distraction for children at busy times in a parent's day and thus reducing time spent in such sedentary activities constitutes a major challenge. A novel strategy might be to provide popular active alternatives to displace sedentary activities [29]. A new generation of active video games ('exer-gaming') such as Sony PlayStation^® ^EyeToy™ and Nintendo Wii™ provide the potential to turn a traditionally sedentary behavior into a physically active one. During these games, players interact physically (using arm, leg, or whole-body movement) with images onscreen in a variety of activities such as sports (e.g., football, boxing, martial arts) and other activities (dancing, washing windows etc). Games are dependent on player movement, and this active component replaces the largely sedentary hand controller of traditional video games whereby button pushing is used to control the game.

Numerous research studies have been conducted to quantify the energy cost of active video games. Without exception, playing active video games have been shown to elicit greater energy expenditure compared to rest and traditional non-active video games, as well as other common sedentary activities such as TV watching [30-32]. Overall, the energy expenditure associated with playing these active video games is similar in intensity to traditional physical activities such as walking, jogging, and cycling, approximately 3–6 Metabolic Equivalents (METS), which are multiples of resting metabolic rate. Recently, active video games have been considered in intervention research. Four small (*n *ranged from 15–60), short-term studies [33-36] have been conducted, of which three reported improvements in physical activity level among children over periods of 4–12 weeks. The fourth study [36] found no effect on physical activity. All studies were of short duration (12 weeks maximum) and thus were unable to provide insight into the sustainability of this intervention and the possible impact on other important variables such as body mass and physical fitness.

Taken together, these findings indicate that active video games are a promising and novel approach to promote physical activity in children. A large, methodologically sound randomized controlled intervention trial is required to determine definitively the long-term effect of active video games on children's physical activity and other outcomes. This paper describes the design and conduct of the eGAME (Electronic Games to Aid Motivation to Exercise) study, which will determine the effects of an active video game intervention over six months on: body mass index (BMI), body weight, percentage body fat, waist circumference, cardio-respiratory fitness, and physical activity levels in overweight NZ children. It was hypothesized that playing 30 minutes of active video games per day over a 6 month period could improve body composition, cardio-respiratory fitness, and physical activity levels in overweight children.

## Methods/Design

### Feasibility studies

The main eGAME study followed a three phase feasibility study conducted in 2005/2006. Phase one consisted of focus groups with children and adults to determine attitudes and preferences to video game play. All children enjoyed active video games, however older children (14 years) preferred traditional non-active games. Adults preferred that children performed physical activity in more traditional outdoor activities but said they would prefer active video game play to the non-active video games. For phase two a laboratory study was conducted to quantify the energy expenditure associated with playing active (Sony EyeToy^®^) and non-active video games [32]. Overall, results showed that energy expenditure was significantly greater in the active video games compared to rest and non-active gaming conditions. The exercise intensity of the active video games ranged between 3.2 and 6.8 child-specific METs.

In phase three a pilot randomized controlled trial (RCT) was conducted to determine the effect of active video game play on physical activity levels [33]. Twenty children aged 10–14 years were randomly assigned to either receive an active video game intervention (n = 10), or no change (control; n = 10). Over the 12 week intervention period, the average time spent in all physical activities (measured by accelerometry) was higher in the active video game intervention group compared to the control group.

### Design

The eGAME study is a standard two-arm parallel RCT. Eligible children are randomized to either an active video games intervention or control (no change) following baseline assessment. The randomization is via a computerized central randomization service and stratified by sex and ethnicity. The study manager and research assistants are responsible for enrolling participants and group assignment. Ethical approval for the study was granted by the Northern Y Ethics Committee (reference: NTY/07/09/099).

### Participants

Children aged 10–14 years living in the greater metropolitan Auckland area, who are overweight (according to the Cole international cut-offs for child obesity) [37], play ≥ two hours of video games per week, and have no contraindications to perform physical activity are eligible to participate in the study. Participants are required to own a PlayStation^® ^2 or 3 gaming console but not the EyeToy™ or Dance Mat technology. Only one child per household is eligible to take part.

### Sample size

A sample size of 330 participants was estimated to provide at least 90% power at 5% level of significance (two-sided) to detect a 0.8 kg/m^2 ^difference in change in BMI from baseline to the end of intervention period between the two groups, assuming a standard deviation (SD) of 2.0 kg/m^2 ^and allowing for 20% loss to follow up. The estimated difference in BMI assumed a difference in weight between groups at the end of intervention of approximately 2 kg with an average height of 1.58 m. This sample size also provides at least 90% power to detect an estimated 3–5 ml/kg/min difference in peak VO_2max _assuming a SD of 7 ml/kg/min. Recruitment of equal numbers of Māori (indigenous people) (n = 110), Pacific, (n = 110) and non-Māori/non-Pacific (n = 110) participants would provide nearly 80% power for the sub-group analysis under the same assumptions.

### Intervention

**Figure 1:**
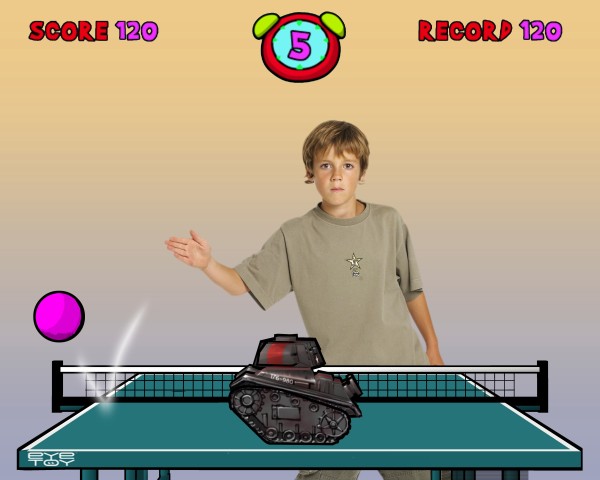
**Example of child playing Sony EyeToy™ active video game**. Reproduced with permission from Sony Computer Entertainment Europe.

The intervention involved an upgrade (hardware and games) of children's existing gaming technology to enable them to play the active video games at home. While various active video game systems exist (e.g., Dance Dance Revolution, Nintento Wii™, Sony EyeToy™) Sony EyeToy™ was used in the current study. This technology uses a USB motion-capture camera to place a picture of the gamer onscreen. The gamer then interacts with the images onscreen (Figure 1). The upgrade consisted of an EyeToy™ camera, dance mat, and a selection of active video games. Relationships were established with Sony Computer Entertainment (SCE) NZ (SCENZ) and Europe (SCEE), and SCEE provided the EyeToy™ cameras and software for the study.

Current physical activity recommendations for children and adolescents are to perform 60-minutes of moderate to vigorous physical activity on most days of the week [38]. In the eGAME study, children were encouraged to meet these recommendations by (1) supplementing periods of inactivity with active video game play; and (2) to substitute periods of traditional inactive video game play with the active version. As compliance with the intervention has been highlighted as an issue in previous active gaming research, several reinforcement strategies were used to maximize compliance in this study. Firstly, the introduction of new games to participants was staggered to maintain interest and motivation (games were given at baseline and again at 12 weeks). Second, intervention participants were reminded at their follow-up assessments to continue playing the games. Children in the control group were asked to continue their normal video game playing and physical activity behavior. At the end of the study, the control group received the active video games upgrade package.

### Procedure

The principle method of recruitment was via schools. Primary (years 1–5), intermediate (years 6–7) and high schools (years 8–13) within the Auckland region were approached to participate in the study. Contact was made with the school principal in the first instance. If the principal agreed to be involved, a school presentation was scheduled, during which children were given brief information about the study. Recruitment also occurred via contacts with local church and community groups, after-school and school holiday programmes, and word of mouth. Interested children provided their contact details to study staff.

Following expression of interest, inclusion and exclusion criteria were assessed in a screening phone call with the child and their parent(s). If eligible, children were posted participant information and informed consent documentation, and booked to attend a baseline assessment. Eligible children attended assessments at a study site close to their place of residence. Four assessment sites were set up in community recreation facilities in the greater Auckland area.

Participants attended assessments at baseline, 12 weeks and 24 weeks, during which they completed a questionnaire assessing key psychological constructs related to physical activity behavior (intention, self-efficacy, perceived competence and enjoyment), had their height, weight and waist circumference measured, underwent bioelectrical impedance analysis (BIA) and completed a field test of physical fitness (20 meter shuttle test). Participants were then given an Actigraph accelerometer to wear for the following seven days to provide an objective assessment of free-living physical activity. During the same period, each participant completed a log-book, detailing their accelerometer wear, their video game (active and nonactive) use and the snack foods they had consumed. Study staff then visited participants at their homes to collect the accelerometer and completed log books.

It was not possible to blind participants to their experimental group allocation. However allocation concealment (up to the point of randomization) was maintained. It was also not possible to blind study staff administering interventions and assessing outcomes to experimental group allocation for pragmatic reasons.

### Measures

#### Anthropometric measures

Anthropometric data were measured using standard practices [39]. Weight was recorded to the nearest 0.1 kilograms using calibrated measurement scales (Salter). Height was measured using a stadiometer (Seca) to the nearest 0.1 cm. Waist circumference data was collected using a flexible non-elastic tape measure. The tape measure was placed around the participant's waist at the level of the umbilicus.

#### Fat mass, fat free mass and percent body fat

BIA was used to estimate fat mass, fat-free mass and percent body fat [40]. Prior to testing, participants were required to lie quietly in a supine position and were kept warm to ensure good circulation. The participant was instructed to lie with their legs slightly apart and hands resting next to the body palms down.

An alcohol wipe was used to remove excess oil and electrodes were placed on the right wrist between the two protruding bones, on the dorsal surface of the right hand 1 cm proximal to the middle knuckle, on the right ankle between the medial and lateral malleoli and on the dorsal surface of the right foot, 1 cm proximal to the metatarsal phalangeal joint of the second toe. The ImpediMed DF50 device was used. The yellow and red leads were connected to the electrodes on the participant's wrist and hand respectively, and the blue and black leads were connected to the electrodes on the participant's ankle and foot respectively. During testing, participants were required to lie still and not talk. Two separate readings were taken from each participant. Output values of impedance, phase, resistance and reactance were recorded.

For participants of Māori or Pacific descent, the Swinburn equation [41] was used to calculate fat mass, fat free mass and percent body fat. For participants who were not of either Māori or Pacific descent, the Lukaski equation [40] was used.

#### Cardiovascular fitness

Cardiovascular fitness was assessed using the 20 meter shuttle test, a standardized field assessment of cardiovascular fitness in children. This test requires participants to carry out a sequence of shuttle runs between two parallel lines positioned 20 meters apart. Each shuttle run is accompanied by a 'beep' audio signal which, when sounded, indicates that the run should have been completed. Those who do not manage this must stop the test and note the number of shuttle runs successfully accomplished and at what level. The timing begins relatively slowly but increases at intervals of approximately one minute. The beep test takes approximately 10 minutes to complete and the output can be used to predict maximal oxygen consumption (VO_2max_) in this age group [42].

#### Self-reported physical activity

Participants completed the Physical Activity Questionnaire for Children (PAQ-C), a validated self-report seven day recall physical activity measure, consisting of nine items that are used to calculate summary activity scores [43]. Items assess physical activity performed at school (physical education, recess, lunchtime), right after school, and at home (organized and recreational). Each PAQ-C statement is scored on a five-point scale with higher scores indicating higher activity levels. A mean score is obtained from each item. The final PAQ-C score is an average of these nine items.

#### Objectively-measured physical activity

Accelerometry was used to assess objectively-measured physical activity. The Actigraph (Model AM7164-2.2C) uniaxial (single-horizontal plane) accelerometer was used, which measures motion in the vertical plane, with movement outside of 'normal' motion being filtered electronically. Piezo-electric transducers and microprocessors detect movement which is converted to digital signals (movement counts).

Participants wore the accelerometer for seven consecutive days following each assessment. They were instructed to wear the monitor on their right hip in the mid-axilla line, during waking hours. Exceptions to this were any activities involving water (water sports, showering etc.), and any contact sports, in which wearing the accelerometer may cause injury to the participant, or damage the monitor. Participants also completed a daily log detailing the time the monitor was first put on and removed.

Because children tend to engage in physical activity in short duration bursts, a 10 second epoch was used and summed to give an activity count per minute [44]. The following cut-off points for METS were used to define light, moderate and vigorous levels of physical activity using the accelerometer: Light = 1.5 – 2.9 METS, moderate = 3 – 5.9 METS, vigorous ≥ 6 METS. For each valid recorded minute, the following equation was used to convert activity counts into equivalent MET values: MET = 2.757 + (0.0015*counts/min) - (0.08957*age) - (0.000038*counts/min*age) [45]. The obtained MET value was then converted into corresponding level of physical activity using the defined cut-off points. A valid accelerometer minute was defined as a recorded minute that does not fall into a sequence of ≥ 20 minutes of missing activity counts. A valid accelerometer day was defined as a recorded day that has a minimum of 600 valid minutes (i.e. 10 hours) [46]. All non-valid days and minutes will be removed from the accelerometer data before analysis. The average daily time spent in light/moderate/vigorous activities is calculated from valid minutes in valid days at each visit (a maximum of 7 days).

#### Psychological variables related to physical activity

Psychological variables were measured to determine their potential mediating effect. Specifically, intention to perform physical activity was assessed using three items [47], which asked participants to rate their level of intention to perform physical activity (e.g. "I intend to take part in regular physical activity most days (at least five) of the next week."). The items were scored using a seven point Likert scale ranging from -3 (completely disagree) to 3 (completely agree). A mean score from the three items is used to give an overall measure of intention.

Physical activity task efficacy was assessed using nine items adapted from the Self Efficacy Scale [48]. Participants were asked to rate their level of confidence to perform physical activity at increasing intensity levels and increasing amounts of time most days of the week. Durations used were 10 minutes, 30 minutes and 60 minutes and intensities were labeled as light, moderate and hard. Participants were provided with descriptions of the intensity levels and examples of types of physical activities at each level. Participants rated their confidence levels on a scale ranging from 0% (I am not confident at all) to 100% (I am completely confident). Scores from these items are summed, and divided by nine to give a mean score for task efficacy. Greater scores indicate greater efficacy to perform physical activity at harder intensities for longer periods of time.

Physical activity barrier efficacy was assessed with six items adapted from the Barriers Efficacy Scale [48]. Items assessed the level of perceived confidence of the participant to perform regular physical activity in the face of a particular barrier (e.g. "If it is bad weather"). Participants rated their confidence levels on a scale ranging from 0% (I am not confident at all) to 100% (I am completely confident). Scores from these items are summed, and divided by six to give a mean score for barrier efficacy. Greater scores indicate greater efficacy to perform physical activity in the face of a variety of barriers.

Perceived competence for physical activity was assessed using the athletic competence six item subscale of the Self-Perception Profile for Children [49]. The participant is provided with two statements and asked to decide which one they are more like (e.g., "Some children do well at sports" vs. "Other children don't do so well"). After deciding between the two alternatives, the participant then decides if the statement is "really true for me" or "sort of true for me". Items are scored on a four point scale, with higher scores indicating more positive self-perceptions. Scores are summed then averaged.

Perceived enjoyment of physical activity was assessed using the 14 item Physical Activity Enjoyment Scale [50], adapted for use in adolescents in 2001 [51]. Participants rate their agreement with statements (e.g., "When I am active I enjoy it") on a five point Likert scale. Scores are summed, with higher scores indicating a higher level of enjoyment.

#### Video game use

During the seven days following each assessment participants completed a daily log detailing the time spent playing video games. A distinction was made between active video games (e.g. EyeToy) and traditional non-active video games (e.g. Xbox). The video game log was pre-tested in the eGAME pilot study [33].

#### Snack food consumption

During the seven days following each assessment participants completed a record of snack foods consumed, which were defined as "something you eat between your main meals (breakfast, lunch and dinner)". The snack food record consisted of pictures of 29 common categories of snack foods and drinks (e.g., pizza, fruit, muesli bars, milk, water etc.). For each food or drink, three pictures were presented showing different snack serving sizes. For example, for the fruit category, the three pictures showed half an apple, a whole apple, and two apples. Participants indicated the number of servings of the respective food and serving size they had consumed on each week day. Each serving size was assigned a caloric value (kJ). The caloric value of all reported snacks is added and then divided by seven days to give average daily total energy consumed from snacks (kJ). The snack food log was developed and pre-tested prior to the study.

### Outcomes

The primary outcome is change in BMI (kg/m^2^, z-score and centile) from baseline to 12 and 24 weeks. The secondary outcomes are change from baseline to 12 and 24 weeks on the following measures: Percent body fat (%), waist circumference (cm), physical fitness measured in VO_2max _(ml/kg/min), PAQ-C score, average daily time spent in light to vigorous activities (minutes), average daily time spent in active video games (minutes) and average daily time spent in non-active video games (minutes). Additional tertiary outcomes include change from baseline to 12 and 24 weeks on the following measures: Average daily time spent in light activities (minutes), moderate activities (minutes) and vigorous activities (minutes); psychological variables intention, self-efficacy, barrier efficacy, perceived competence, perceived enjoyment; and average daily total energy consumed from snacks (kJ).

### Statistical analysis

Statistical analyses will be performed using SAS version 9.1.3 (SAS Institute Inc. Cary NC) and R version 2.8.1 (R Foundations for Statistical Computing). All statistical tests will be two-tailed and a 5% significance level maintained throughout the analyses. All treatment evaluations will be performed on the principle of Intention to Treat (ITT). Repeated measures analysis using the mixed model will be carried out to evaluate the effect of intervention on change from baseline measures at 12 and 24 weeks, adjusting for baseline outcome measure, age (in years), sex (male/female), and ethnicity (Māori/Pacific/nMnP). With roughly equal number of Māori, Pacific and non-Māori non-Pacific participants, appropriate sub-group analysis will be carried out for each ethnicity if there is a significant overall treatment effect.

## Discussion

A number of reflections on study design and conduct arising from this RCT are likely to be of interest to those undertaking similar work.

### Determining the feasibility of conducting such a trial

The feasibility studies [32,33] were critical to inform the design of the main study. The focus groups gave insight into the video game preferences of children; in particular, it was identified that older children preferred traditional non-active gaming, and thus were unlikely to be complaint to an active video games intervention. The researchers therefore tailored the intervention to an appropriate age group (10–14 years) for the main trial. Also, parents generally supported the concept of active video gaming, and would therefore be more likely to promote home use of the games. The energy expenditure study provided information specific to the gaming system of interest (Sony EyeToy™), gave insight into whether the active games were sufficiently vigorous such that they could be used as a physical activity intervention, and gave an indication of how much game play may be used to cause an appreciable change in body composition.

The pilot RCT identified a myriad of issues relevant to the design and conduct of the main trial. Potential compliance problems were identified; the effect of the intervention decreased from six to 12 weeks, which raised issues of sustained interest in the intervention. For this reason, the introduction of new games was staggered throughout the intervention in the main trial to maintain interest. The pilot RCT also allowed the researchers the opportunity to develop and test the study materials and protocols on a small scale prior to the main trial. In particular, issues relating to management of the accelerometer data were resolved, such as the appropriate epoch and data reduction protocols, and systems put in place for the management of larger amounts of data. A new snack log was developed that was more appropriate to the age group than the instrument used in the pilot trial. Feasibility of recruitment processes was tested; recruitment was 100% for all three phases, with good representation by Māori and Pacific peoples.

### Developing a relationship with a corporate

Working relationships were developed with SCEE and SCENZ. SCEE provided Sony EyeToy cameras and games for study participants. However, their involvement posed a potential conflict of interest; therefore, the roles and responsibilities of both the corporate and academic parties were clearly outlined in a formal agreement prior to the start of the study. Funding for the study was secured from a national health research funding body, and SCEE did not contribute to the design, conduct or analysis of the research.

### Working with schools to conduct research

The primary mode of recruitment has been through schools. Therefore, developing and maintaining an effective working relationship with schools is crucial to the success of the project. The participation of schools in research is voluntary and in the past has often involved significant disruption to the school timetable and the requirement of staff time to help co-ordinate data collection. Furthermore, there is no common method to approach schools, which has resulted in school principals fielding scores of requests from researchers weekly. This has resulted in a somewhat strained relationship between schools and the academic community and an increasing reluctance for schools to be involved in research. The eGAME recruitment strategy sought to minimize disruption to school processes. With the principal's permission, a 5–10 minute presentation was scheduled during a regular school assembly slot. In this presentation, a brief outline of the study was given and interested students wrote their contact details on forms distributed to classes. Approximately seven days later, these forms were collected from the school. Potential participants and their parents were then contacted outside of school hours for screening. All assessments took place in the evening or weekend so as not to disrupt school attendance. Researchers seeking to undertake research with school involvement should aim to minimize the use of school time and resources where possible. Furthermore, researchers should keep schools up to date with relevant information and the results of the trial once completed.

### Recruitment of indigenous and ethnic minority populations

Researchers have typically struggled to recruit sufficient participants from indigenous and ethnic minority populations to allow for sub-analyses to be undertaken by ethnicity. The eGAME study aims to recruit equal numbers of Māori, Pacific, and nMnP participants, and proactive steps were taken to maximize recruitment in Māori and Pacific populations.

Consultation with Māori and Pacific communities during the focus groups provided useful feedback on design of the main study. A specialized Māori/Pacific recruiter was contracted to assist with recruitment of these populations and consult on cultural issues relevant to the conduct of the research. As a result, additional recruitment sites were set up in the greater Auckland area where a large number of Māori and Pacific resided, to improve access of these populations to the study. Furthermore, some study processes were modified to improve cultural appropriateness. For example, at one study site the screening phone call was changed to a face-to-face visit between study staff and the potential participant and their family, the preferred mode of communication for Māori and Pacific populations. The Māori/Pacific recruiter maintained regular contact with participants throughout the course of their involvement with study. Despite these steps, recruitment of Māori and Pacific children remains a challenge. At the time of submission of this manuscript, the eGAME sample comprised 17% Māori and 27% Pacific participants, below the target of 33% for each.

### Potential to encourage less healthy behaviors and increase disparity

During the conceptualization of the study, there was particular concern that the intervention would act to promote additional video game play in participants, including heightened use of traditional sedentary games. However, results from the pilot study indicated that children receiving the active video games intervention spent less total time playing all electronic games compared to those in the control group [33]. In the eGAME trial, intervention participants were instructed to substitute the time spent playing non-active video games with active games, rather than increase their overall video game play. Furthermore, participants were required to already be owners and regular users of video game console technology to be eligible for the study, so that video game use was not promoted in those who did not currently own them. Researchers were sensitive to the potential that the video console ownership criteria could increase disparities, as ownership may differ by ethnic group. However, market research statistics indicated this was not the case; video game console ownership in 2005 was higher among Māori (46%) than non-Māori (31%–44%) [52].

### Methodology issues in pragmatic trials

The eGAME study is an example of a pragmatic trial, in which the study is designed to maximize the applicability of the trial's results to usual care settings [53]. The criteria for selection were broad and aimed to be as inclusive as possible to reduce homogeneity of the sample population and improve the generalizability of results.

The eGAME study aims to examine the effectiveness of a video game intervention that is widely commercially available. Furthermore, the context of the trial is the participant's own home environment where video game use tends to occur. Participants in the intervention group were asked to report on their game play, though this was not directly observed. Therefore, control of the intervention was not stringent and the implementation of the intervention was slightly different for each participant with regards to frequency and duration of play. Nevertheless, this will provide researchers with an indication of the usefulness of this intervention in a naturalistic setting, and frequency and duration of self-directed play are important outcomes in themselves.

### Implications for future studies

Investigation into the feasibility of the proposed intervention, as well as robust testing of proposed study procedures is a critical step prior to implementation of a large-scale trial. These preliminary steps will likely enhance intervention effectiveness, identify potential issues specific to the context of the research, and help to ensure sufficient numbers of participants are able to be recruited to detect an effect. Partnering with industry can be mutually beneficial, though researchers must remain vigilant to potential conflicts of interest and be transparent as to where the involvement of the commercial partner lies. Results of the eGAME study are due in early 2010, and will be disseminated to relevant community and government organizations, as well as being published in academic journals. In the meantime, it is hoped that the issues discussed provide guidance to those undertaking similar trials with children.

## Competing interests

The relationship between the authors and SCEE and SCENZ has been outlined. The authors declare that they have no competing interests.

## Authors' contributions

Principal responsibility for study design and conduct was assumed by RM. CNM, AJ, AR, HP, MH and DS contributed to the study design. YJ and SVH undertook sample size calculations and designed the statistical analysis plan. LF was involved in the study design, data collection and overall project management. LF and RM drafted the manuscript. All authors read and commented on drafts and approved the final manuscript.

## Pre-publication history

The pre-publication history for this paper can be accessed here:



## References

[B1] Wang Y, Lobstein T (2006). Worldwide trends in childhood overweight and obesity. International Journal of Pediatric Obesity.

[B2] Ministry of Health (2003). NZ food NZ children: key results of the 2002 national children's nutrition survey.

[B3] Prentice AM, Jebb SA (1995). Obesity in Britain: gluttony or sloth?. British Medical Journal.

[B4] Wake M, Hesketh K, Waters E (2003). Television, computer use and body mass index in Australian primary school children. Journal of Paediatric Child Health.

[B5] International Obesity Taskforce and European Association for the Study of Obesity (2002). Obesity in Europe. The case for action.

[B6] Proctor MH, Moore LL, Gao D, Cupples LA, Bradlee ML, Hood MY, Ellison RC (2003). Television viewing and change in body fat from preschool to early adolescence: The Framingham Children's Study. International Journal of Obesity & Related Metabolic Disorders.

[B7] Pardee PE, Norman GJ, Lustig RH, Preudhomme D, Schwimmer JB (2007). Television viewing and hypertension in obese children. American Journal of Preventive Medicine.

[B8] Statistics New Zealand (2001). 2000/01 household economic survey.

[B9] Nielsen AC (2004). How long do we spend watching television?.

[B10] Hancox RJ, Milne BJ, Poulton R (2004). Association between child and adolescent television viewing and adult health: a longitudinal birth cohort study. The Lancet.

[B11] Pratchett R (2005). Gamers in the UK: Digital play, digital life styles.

[B12] Nielsen AC (2005). National Readership Survey and Panorama.

[B13] Kaiser Family Foundation (2002). Key Facts: Children and video games.

[B14] Scragg R, Quigley R, Taylor R (2006). Does watching TV contribute to increased body weight and obesity in children?.

[B15] Summerbell CD, Waters E, Edmunds LD, Kelly S, Brown T, Campbell KJ (2005). Interventions for preventing obesity in children, (Cochrane Review). The Cochrane Library, 2005 The Cochrane Database of Systematic Reviews.

[B16] Summerbell CD, Waters E, Edmunds LD, Kelly S, Brown T, Campbell KJ (2005). Interventions for preventing obesity in children, (Cochrane Review). The Cochrane Library, 2005 The Cochrane Database of Systematic Reviews.

[B17] Caballero B, Clay T, Davis SM, Ethelbah E, Holy Rock B, Lohman T, Norman J, Story M, Stone EJ, Stephenson L, Stevens J (2003). Pathways: a school-based randomized controlled trial for the prevention of obesity in American Indian schoolchildren. American Journal of Clinical Nutrition.

[B18] Donnelly JE, Jacobsen DJ, Whatley JE, Hill JO, Swift LL, Cherrington A, Polk B, Tran ZV, Reed G (1996). Nutrition and physical activity program to attenuate obesity and promote physical and metabolic fitness in elementary school children. Obesity Research.

[B19] Gortmaker SL, Peterson K, Wiecha J, Sobol AM, Dixit S, Fox MK, Laird N (1999). Reducing obesity via a school-based inter-disciplinary intervention among youth: planet health. Archives of Pediatric and Adolescent Medicine.

[B20] Mueller MJ, Asbeck I, Mast M, Lagnaese L, Grund A (2001). Prevention of Obesity – more than an intention. Concept and first results of the Kiel Obesity Prevention Study (KOPS). International Journal of Obesity.

[B21] Sahota P, Rudolf MCJ, Dixey R, Hill AJ, Barth JH, Cade J (2001). Evaluation of implementation and effect of primary school based intervention to reduce risk factors for obesity. British Medical Journal.

[B22] Warren JM, Henry CJK, Lightowler HJ, Bradshaw SM, Perwaiz S (2003). Evaluation of a pilot school programme aimed at the prevention of obesity in children. Health Promotion International.

[B23] Mo-Suwan L, Pongprapai S, Junjana C, Peutpaiboon A (1998). Effects of a controlled trial of a school-based exercise program on the obesity indexes of preschool children. American Journal of Clinical Nutrition.

[B24] Sallis JF, McKenzie TL, Alcaraz JE, Kolody B, Hovell MF, Nader PR (1993). Project SPARK. Effects of physical education on adiposity in children. Annals of the New York Academy of Sciences.

[B25] R Flores (1995). Dance for Health: Improving fitness in African American and Hispanic Adolescents. Public Health Reports.

[B26] Neumark-Sztainer D, Story M, Hannan PJ, Rex J (2003). New Moves: a school-based obesity prevention program for adolescent girls. Preventive Medicine.

[B27] Pangrazi RP, Beighle A, Vehige T, Vack C (2003). Impact of Promoting Lifestyle Activity for Youth (PLAY) on children's physical activity level. Journal of School Health.

[B28] Robinson TN (1999). Reducing television's viewing to prevent obesity: A randomized controlled trial. Journal of the American Medical Association.

[B29] Dietz WH, Gortmaker SL (2001). Preventing obesity in children and adolescents. Annual Review of Public Health.

[B30] Straker L, Abbott R (2007). Effect of screen-based media on energy expenditure and heart rate in 9- to 12-year-old children. Pediatric Exercise Science.

[B31] Graves L, Stratton G, Ridgers ND, Cable NT (2007). Comparison of energy expenditure in adolescents when playing new generation and sedentary computer games: cross sectional study. BMJ.

[B32] Maddison R, Ni Mhurchu C, Jull A, Jiang Y, Prapavessis H, Rodgers A (2007). Energy expended playing video console games: an opportunity to increase children's physical activity?. Pediatric Exercise Science.

[B33] Ni Mhurchu C, Maddison R, Jiang Y, Jull A, Prapavessis H, Rodgers A (2008). Couch potatoes to jumping beans: A pilot study of the effect of active video games on physical activity in children. International Journal of Behavioral Nutrition and Physical Activity.

[B34] Palmeira AL, Martines SS, Fonseca H, Veloso S, Cunha L, Neves R, Ravasco P (2008). Impact of motion matching video-games on one-month's physical activity levels of overweight adolescents. 7th Annual Conference of the International Society of Behavioral Nutrition and Physical Activity.

[B35] Maloney AE, Bethea TC, Kelsey KS, Marks JT, Paez S, Rosenberg AM, Catellier DJ, Hamer RM, Sikich L (2008). A Pilot of a Video Game (DDR) to Promote Physical Activity and Decrease Sedentary Screen Time. Obesity.

[B36] Murphy EC (2007). The Effect of Aerobic Exercise on Endothelial Dysfunction in Overweight Children. School of Medicine.

[B37] Cole TJ, Bellizzi MC, Flegal KM, Dietz WH (2000). Establishing a standard definition for child overweight and obesity worldwide: international survey. British Medical Journal.

[B38] Australian Government Department of Health and Ageing Australia's Physical Activity Recommendations for Childrenand Young People. http://www.health.gov.au/internet/main/publishing.nsf/Content/health-pubhlth-strateg-active-recommend.htm.

[B39] McArdle WD, Katch FI, Katch VL (1991). Exercise Physiology: Energy, Nutrition, and Human Performance.

[B40] Lukaski HC, Bolonchuk WW, Hall CB, Siders WA (1986). Validation of tetrapolar bioelectrical impedance method to assess human body composition. Journal of Applied Physiology.

[B41] Swinburn BA, Ley SJ, Carmichael HE, Plank LD (1999). Body size and composition in Polynesians. International Journal of Obesity.

[B42] Ramsbottom R, Brewer J, Williams C (1988). A progressive shuttle run test to estimate maximal oxygen uptake. British Journal of Sports Medicine.

[B43] Crocker PR, Bailey DA, Faulkner RA, Kowalski KC, McGrath R (1997). Measuring general levels of physical activity: preliminary evidence for the Physical Activity Questionnaire for Older Children. Med Sci Sports Exerc.

[B44] Ward DS, Evenson KR, Vaughn A, Rodgers AB, Troiano RP (2005). Accelerometer use in physical activity: Best practices and research recommendations. Medicine & Science in Sports & Exercise.

[B45] Freedson P, Pober D, Janz KF (2005). Calibration of accelerometer output for children. Medicine & Science in Sports & Exercise.

[B46] Masse LC, Fuemmeler BF, Anderson CB, Matthews CE, Trost SG, Catellier DJ, Treuth M (2005). Accelerometer data reduction: A comparison of four reduction algorithms on select outcome variables. Medicine & Science in Sports & Exercise.

[B47] Ajzen I (2002). Constructing a TPB questionnaire: Conceptual and Methodological Considerations. http://socgeo.ruhosting.nl/html/files/spatbeh/tpb.measurement.pdf.

[B48] McAuley E, Mihalko SL, Duda JL (1998). Measuring exercise-related self-efficacy. Advances in sport and exercise psychology measurement.

[B49] Harter S (1985). Manual for the Self-Perception Profile for Children.

[B50] Kendzierski D, DeCarlo KJ (1991). Physical Activity Enjoyment Scale: Two Validation Studies. Journal of Sport and Exercise Psychology.

[B51] Motl RW, Dishman RK, Saunders R, Dowda M, Felton G, Pate RR (2001). Measuring enjoyment of physical activity in adolescent girls. American Journal of Preventive Medicine.

[B52] Nielsen AC (2005). National Readership Survey and Panorama.

[B53] Zwarenstein M, Treweek S, Gagnier JJ, Altman DG, Tunis S, Haynes B, Oxman AD, Moher D (2008). Improving the reporting of pragmatic trials: an extension of the CONSORT statement. BMJ.

